# Pennsylvania State Dental Society

**Published:** 1897-08

**Authors:** 


### PENNSYLVANIA STATE DENTAL SOCIETY.

The meeting of the Pennsylvania State Dental Society, held at Glen Summit, Pa., July 6, 7, and 8, had a very large attendance, and in many respects was the most important gathering held in the State for some years.

The new dental law passed by the Legislature, and to which the governor has since appended his signature, made the selection of the Board of Examiners an important part of the work. This was accomplished without friction, and it is anticipated that the governor will make the appointments, at an early date, from the names selected by the Society.

The law, as adopted, is one of the best upon the statutes of the several States, and obviates some of the serious objections found in many of the crude productions elsewhere formulated.

The unfortunate difficulty that this Society met with at the session of 1896 was thoroughly examined into, and, it is believed, the conclusions of the committee, having it in charge, will amicably settle all the contentions that have disturbed dental professional circles for the past year. The conclusions of the committee will be found under the proper heading.

This meeting enforces previous conclusions of the writer, that State organizations are drifting more and more into legislative bodies, and it is hopeless to expect extended scientific results in that direction. The present meeting was mainly occupied with this character of work. The few papers read were excellent, but they were principally relegated to the last session, which is neither complimentary to the writers or satisfactory to the audience. Unless some improvements are made in the direction of essays, the attendance at future meetings of the Society will be confined mainly to those interested in the legislative work of the organization. Such a result should be a matter of regret, for, with proper care in the arrangement of business, it might be avoided in the future.

